# Downregulation of Glutathione Biosynthesis Contributes to Oxidative Stress and Liver Dysfunction in Acute Kidney Injury

**DOI:** 10.1155/2016/9707292

**Published:** 2016-10-30

**Authors:** Yue Shang, Yaw L. Siow, Cara K. Isaak, Karmin O

**Affiliations:** ^1^St. Boniface Hospital Research Centre, Winnipeg, MB, Canada; ^2^Department of Animal Science, University of Manitoba, Winnipeg, MB, Canada; ^3^Department of Physiology and Pathophysiology, University of Manitoba, Winnipeg, MB, Canada; ^4^Agriculture and Agri-Food Canada, Winnipeg, MB, Canada

## Abstract

Ischemia-reperfusion is a common cause for acute kidney injury and can lead to distant organ dysfunction. Glutathione is a major endogenous antioxidant and its depletion directly correlates to ischemia-reperfusion injury. The liver has high capacity for producing glutathione and is a key organ in modulating local and systemic redox balance. In the present study, we investigated the mechanism by which kidney ischemia-reperfusion led to glutathione depletion and oxidative stress. The left kidney of Sprague-Dawley rats was subjected to 45 min ischemia followed by 6 h reperfusion. Ischemia-reperfusion impaired kidney and liver function. This was accompanied by a decrease in glutathione levels in the liver and plasma and increased hepatic lipid peroxidation and plasma homocysteine levels. Ischemia-reperfusion caused a significant decrease in mRNA and protein levels of hepatic glutamate-cysteine ligase mediated through the inhibition of transcription factor Nrf2. Ischemia-reperfusion inhibited hepatic expression of cystathionine *γ*-lyase, an enzyme responsible for producing cysteine (an essential precursor for glutathione synthesis) through the transsulfuration pathway. These results suggest that inhibition of glutamate-cysteine ligase expression and downregulation of the transsulfuration pathway lead to reduced hepatic glutathione biosynthesis and elevation of plasma homocysteine levels, which, in turn, may contribute to oxidative stress and distant organ injury during renal ischemia-reperfusion.

## 1. Introduction

Ischemia-reperfusion (IR) is one of the common causes for acute kidney injury (AKI) [1–3]. AKI often leads to multiorgan dysfunction known as distant organ injury [4, 5]. Renal IR occurs in many clinical situations such as renal transplantation and other surgery procedures or as comorbidity in critically ill patients. However, mechanisms of AKI-induced distant organ injury are not well understood. Clinically, distant organ injury potentiates the already high rate of AKI-associated morbidity and mortality [6, 7]. In animal models, AKI is shown to cause injury of multiple organs including brain, heart, intestine, liver, and lung [4, 8–13].

**Figure 1:**
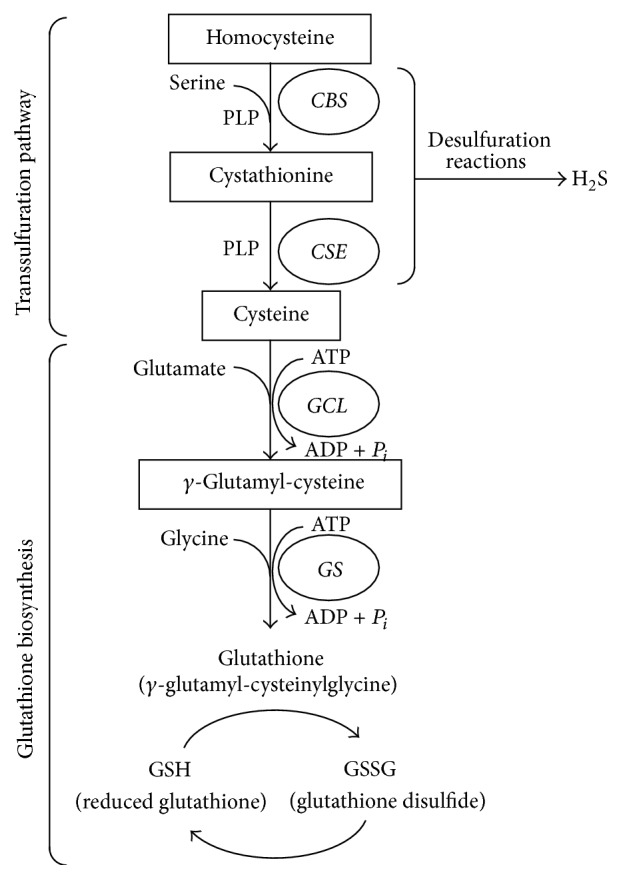
Transsulfuration pathway and glutathione biosynthetic pathway. The transsulfuration pathway metabolizes homocysteine to cysteine. In this pathway, cystathionine *β*-synthase (*CBS*) catalyzes the initial reaction, in which homocysteine is condensed with serine to form cystathionine. Cystathionine is subsequently metabolized to cysteine by the second enzyme, cystathionine *γ*-lyase (*CSE*). Both CBS and CSE are pyridoxal-5′-phosphate- (PLP-) dependent enzymes. The tripeptide glutathione is synthesized from glutamate, cysteine, and glycine. Glutamate-cysteine ligase (*GCL*) catalyzes the rate-limiting reaction by converting cysteine and glutamate to *γ*-glutamyl-cysteine. The second enzyme, glutathione synthase (*GS*), catalyzes the reaction of *γ*-glutamyl-cysteine and glycine to form glutathione. The equilibrium between reduced (GSH) and oxidized (GSSG) glutathione reflects the redox potential of a given tissue, with lower GSH : GSSG ratios being indicative of oxidative stress. Alternatively, CBS and CSE also mediate the desulfuration reactions which lead to hydrogen sulfide (H_2_S) synthesis.

The pathogenesis of IR injury is multifaceted and oxidative stress is considered as one of the important mechanisms responsible for local as well as distant organ injury. Impaired antioxidant defense and/or overproduction of reactive oxygen species (ROS) can lead to oxidative stress. Glutathione is a thiol-containing tripeptide that serves as a major endogenous nonenzymatic antioxidant against oxidative stress [14, 15]. The liver has high glutathione levels due to its efficient synthesis [14–18]. Impaired liver function and metabolism have been found in animal models with ischemic AKI [19–22]. It has been shown that renal IR causes depletion of hepatic glutathione, while administration of this antioxidant effectively attenuates oxidative stress and improves liver function [22]. However, the mechanism by which ischemic AKI elicits hepatic glutathione depletion is not well understood. Glutathione is synthesized by two enzymes, namely, glutamate-cysteine ligase (EC 6.3.2.2) and glutathione synthase (EC 6.3.2.3) (Figure 1) [17]. Glutamate-cysteine ligase is composed of a catalytic subunit (*Gclc*) and a modifier subunit (*Gclm*). This enzyme catalyzes the rate-limiting reaction by converting cysteine and glutamate to *γ*-glutamyl-cysteine. The second enzyme, glutathione synthase, catalyzes the reaction of *γ*-glutamyl-cysteine and glycine to form glutathione. Nuclear factor-erythroid 2-related factor-2 (Nrf2) is a key transcription factor involved in cellular responses against oxidative stress. It regulates the expression of many antioxidant proteins and enzymes [17, 23, 24]. Activation of Nrf2 induces gene expression of cytoprotective enzymes including those that are responsible for glutathione synthesis [23, 25–27]. Under normal conditions, Nrf2 is retained in the cytoplasm through binding to a repressor protein named Kelch-like ECH-associated protein 1 (Keap1). Upon stimulation, Nrf2 is dissociated from Keap1 and translocated into the nucleus, where it binds to the promoter regions of the target genes [23, 24]. Nuclear translocation of Nrf2 can be stimulated by hydrogen sulfide through S-sulfhydration of Keap1 [28, 29]. It has been shown that deficiency of Nrf2 leads to increased oxidative stress, renal damage and hepatic damage, and inflammatory responses [23, 24, 26]. Although the protective effect of Nrf2 through upregulation of antioxidant enzymes against IR or toxin induced organ injury has been implicated [24, 30, 31], its role in AKI-induced distant organ injury is not clear.

The availability of cysteine is another important determinant in modulating glutathione homeostasis as this sulfur-containing amino acid is an essential precursor for glutathione biosynthesis. Aside from dietary intake or endogenous protein degradation, the transsulfuration pathway is the only source of* de novo* synthesized cysteine in mammalian cells (Figure 1) [17, 32, 33]. In the transsulfuration pathway, cystathionine *β*-synthase (*CBS*, EC 4.2.1.22) catalyzes the initial reaction in which homocysteine is condensed with serine to form cystathionine. Cystathionine is subsequently metabolized to cysteine by another enzyme, cystathionine *γ*-lyase (*CSE*, EC 4.4.1.1). By generating cysteine, the transsulfuration pathway provides the rate-limiting amino acid for cellular glutathione biosynthesis [33–37]. This may be particularly important in the liver as 50% of the cellular glutathione pool in hepatocytes is derived from the transsulfuration pathway [14]. The transsulfuration pathway enzymes are widely present in mammalian tissues such as liver, kidney, intestine, heart, aorta, brain, lungs, and intestine, with liver having high enzyme activity of both* CBS* and* CSE* [14, 33–38]. Our recent study has shown that IR downregulates the expression of transsulfuration enzymes in the kidney, leading to increased oxidative stress and inflammatory response [39]. Although liver oxidative stress has been reported in ischemic AKI, the mechanism by which renal IR impairs hepatic glutathione production is not well understood. As glutathione is the major endogenous antioxidant and the liver is the key organ for its generation, its depletion could lead to local and systemic oxidative stress. In the present study, we investigated the mechanism by which renal IR caused downregulation of hepatic glutathione biosynthesis and oxidative stress.

## 2. Materials and Methods

### 2.1. Animal Model

Sprague-Dawley male rats (250–300 g, 7-8 weeks old) were fed a commercial diet (Prolab® RMH 3000, 5P00) containing 0.40% of cysteine and 0.58% of methionine (LabDiet, St. Louis, MO) prior to surgery. Rats were anesthetised by 3% isoflurane/oxygen gas. Renal ischemia was induced by clamping the left kidney pedicle for 45 min as described in our previous studies [40–42]. At the end of ischemia, the rats were subjected to 6 h of reperfusion by removal of the clamp and right nephrectomy. During the surgery, rats were kept on a heat pad and 1-2% isoflurane/oxygen gas was maintained via inhalation. As a control (sham-operated), rats were subjected to the same surgical procedure without inducing ischemia and were sacrificed at the corresponding time point. A blood sample was collected and centrifuged at 3000 ×g for 20 min for plasma preparation. Plasma creatinine, alanine aminotransferase, and aspartate aminotransferase were measured using the Cobas C111 analyzer (Roche, Laval, QC). All procedures were performed in accordance with the Guide to the Care and Use of Experimental Animals published by the Canadian Council on Animal Care and approved by the University of Manitoba Protocol Management and Review Committee.

### 2.2. Biochemical Analyses

Reduced (GSH) and oxidized (GSSG) glutathione in the plasma and liver were measured by a spectrophotometric detection method [39, 43, 44]. A ratio of GSH and GSSG was determined as an indicator of redox potential. The degree of lipid peroxidation in the liver tissue was determined by measuring malondialdehyde (MDA) levels with thiobarbituric acid reactive substances (TBARS) assay [45, 46]. Cysteine content in the liver was measured by ion exchange chromatographic method using an amino acid analyzer S430 (Sykam, Eresing, Germany). Hydrogen sulfide (H_2_S) production was measured based on a spectrophotometric detection method of Stipanuk and Beck [37] as described in our previous studies [39, 44, 47].

### 2.3. Transfection of HepG2 Cells with Nrf2 siRNA

HepG2 cells (American Type Culture Collection, MA, a cell line derived from human hepatoblastoma) were transfected with Nrf2 siRNA oligonucleotides (Life Technologies, Carlsbad, CA) or RNAi negative control consisting of scrambled oligonucleotides using Lipofectamine 2000 (Invitrogen, Carlsbad, CA) as transfection reagent. After 6 h incubation, the medium was replaced with HyClone™ Dulbecco's Modified Eagle Medium (containing L-cystine 2HCl, 62.57 mg/L, and L-methionine, 30.00 mg/L) supplemented with 10% fetal bovine serum and incubated for another 48 h. The mRNA of Nrf2 and glutamate-cysteine ligase subunits (*Gclc* and* Gclm*) was determined.

### 2.4. Real-Time Polymerase Chain Reaction (PCR) Analysis

Total RNAs were isolated from the liver tissue with TRIzol reagent (Invitrogen, Carlsbad, CA). The mRNA of glutamate-cysteine ligase catalytic subunit (*Gclc*) and modifier subunit (Gclm), glutathione synthase, and enzymes in the transsulfuration pathway (*CBS* and* CSE*) and Nrf2 was determined by real-time PCR analysis using the iQ5 real-time PCR detection system (Bio-Rad) and normalized with *β*-actin [39, 41, 44]. The primers (Invitrogen) used for rat mRNA measurement were as follows:* Gclc* (124 bp), 5′-GCCCAATTGTTATGGCTTTG-3′ (forward) and 5′-AGTCCTCTCTCCTCCCGTGT-3′ (reverse) (GenBank accession number: NM_012815);* Gclm* (114 bp), 5′-CGAGGAGCTTCGAGACTGTAT-3′ (forward) and 5′-ACTGCATGGGACATGGTACA-3′ (reverse) (GenBank accession number: NM_017305); glutathione synthase (182 bp), 5′-ACAACGAGCGAGTTGGGAT-3′ and 5′-TGAGGGGAAGAGCGTGAATG-3′ (reverse) (GenBank accession number: NM_012962); rat CBS (148 bp), 5′-TCGTGATGCCAGAGAAGATG-3′ (forward) and 5′-TTGGGGATTTCGTTCTTCAG-3′ (reverse) (GenBank accession number: NM_012522); CSE (150 bp), 5′-GTATGGAGGCACCAACAGGT-3′ (forward) and 5′-GTTGGGTTTGTGGGTGTTTC-3′ (reverse) (GenBank accession number: NM_017074); and ß-actin (198 bp), 5′-ACAACCTTCTTGCAGCTCCTC-3′ (forward) and 5′-GACCCATACCCACCATCACA-3′ (reverse) (GenBank accession number: NM_031144). Primers (Invitrogen) used for human mRNA measurement were as follows: Nrf2 (106 bp), 5′-AGTGGATCTGCCAACTACTC-3′ (forward) and 5′-CATCTACAAACGGGAATGTCTG-3′ (reverse) (GenBank accession number: NM_006164);* Gclc* (105 bp), 5′-TACAGTTGAGGCCAACATGC-3′ (forward) and 5′-CTTGTTAAGGTACTGGGAAATGAAG-3′ (reverse) (GenBank accession number: NM_001197115);* Gclm* (102 bp), 5′-GTTCAGTCCTTGGAGTTGCACA-3′ (forward) and 5′-CCCAGTAAGGCTGTAAATGCTC-3′ (reverse) (GenBank accession number: NM_002061); and ß-actin (95 bp), 5′-AGATCAAGATCATTGCTCCTCCT-3′ (forward) and 5′-GATCCACATCTGCTGGAAGG-3′ (reverse) (GenBank accession number: NM_001101).

### 2.5. Western Immunoblotting Analysis

The protein levels of hepatic glutamate-cysteine ligase catalytic (*Gclc*) and modifier (*Gclm*) subunits, glutathione synthase, and enzymes in the transsulfuration pathway (*CBS* and* CSE*) were determined by Western immunoblotting analysis. Total proteins (10 *μ*g) were separated by electrophoresis in 10% SDS polyacrylamide gels. Proteins in the gel were transferred to a nitrocellulose membrane. The membrane was probed with rabbit anti-Gclc monoclonal (1 : 2000, Abcam, Inc., Toronto, Canada), rabbit anti-Gclm monoclonal (1 : 2000, Abcam, Inc., Toronto, Canada), rabbit anti-glutathione synthase monoclonal (1 : 4,000; Abcam, Inc., Toronto, Canada), mouse anti-*CBS* monoclonal (1 : 4000, Abnova Corporation, Taipei, Taiwan), or rabbit anti-*CSE* monoclonal (1 : 4000, GeneTex, Irvine, CA) antibodies for total liver proteins. Nuclear proteins (90 *μ*g) were used to determine Nrf2 protein with anti-rabbit Nrf2 monoclonal antibodies (1 : 500, Abcam, Inc., Toronto, Canada). HRP-conjugated anti-mouse or anti-rabbit IgG antibodies (Cell Signaling Technology, Danvers, MA) were used as the secondary antibodies (1 : 2000). The corresponding protein bands were visualized using enhanced chemiluminescence reagents and analyzed with a gel documentation system (Bio-Rad Gel Doc1000). To confirm the equal loading of proteins for each sample, the same membranes were probed with mouse anti-ß-actin monoclonal antibodies (1 : 5000, Cell Signaling Technology, Danvers, MA) or rabbit anti-Histone H3 monoclonal antibodies (1 : 500, Santa Cruz Biotechnology, Inc., Santa Cruz, CA).

### 2.6. Statistical Analysis

Results were analyzed using two-tailed Student's* t*-test. *p* values less than 0.05 were considered statistically significant.

## 3. Results

### 3.1. Renal Ischemia-Reperfusion Impaired Kidney and Liver Function

**Figure 2:**
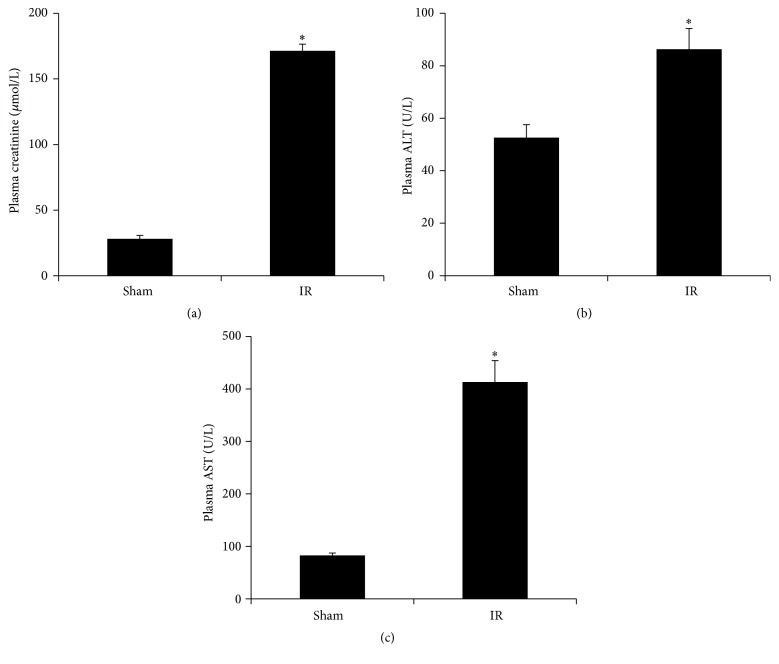
Effect of kidney ischemia-reperfusion on kidney and liver function. The left kidney of rats was subjected to 45 min ischemia followed by 6 h reperfusion (IR). As a control, rats were subjected to a sham operation without inducing ischemia (Sham). Plasma creatinine (a), alanine aminotransferase (ALT) (b), and aspartate aminotransferase (AST) (c) were measured. Results are expressed as mean ± SE (*n* = 4 for each group). ^*∗*^ *p* < 0.05 when compared with the value obtained from the sham-operated group.

**Figure 3:**
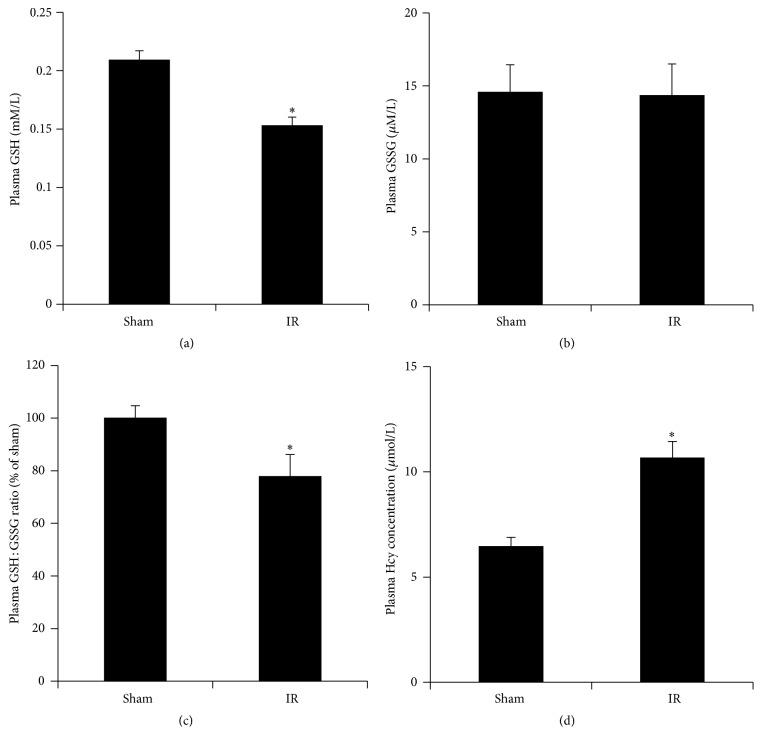
Effect of kidney ischemia-reperfusion on glutathione and homocysteine levels in the plasma. Plasma reduced glutathione (GSH) (a), oxidized glutathione (GSSG) (b), a ratio of reduced glutathione (GSH) to oxidized glutathione (GSSG) (c), and plasma homocysteine (d) were measured in rats subjected to renal IR or sham operation. Results are expressed as mean ± SE (*n* = 4 for each group). ^*∗*^ *p* < 0.05 when compared with the value obtained from the sham-operated group.

Renal IR (45 min ischemia followed by 6 h reperfusion) resulted in a significant elevation of plasma creatinine level (Figure 2(a)), indicating that kidney function was impaired. Renal IR also caused a marked increase in plasma alanine aminotransferase (ALT) and aspartate aminotransferase (AST) levels (Figures 2(b) and 2(c)), suggesting that IR not only impaired renal function but also caused liver injury. Renal IR significantly decreased the reduced glutathione (GSH) levels (Figure 3(a)) but did not change the oxidized glutathione (GSSG) levels (Figure 3(b)), leading to a low ratio of GSH to GSSG in the plasma (Figure 3(c)). There was a significant elevation of plasma homocysteine level in rats subjected to renal IR (Figure 3(d)).

### 3.2. Renal Ischemia-Reperfusion Reduced Glutathione Synthesis and Induced Oxidative Stress in the Liver

**Figure 4:**
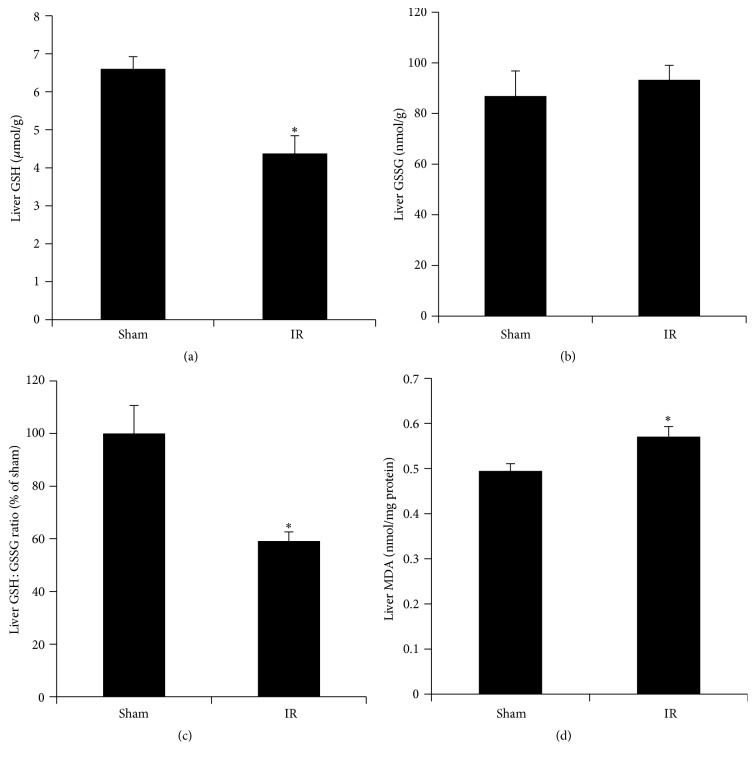
Effect of kidney ischemia-reperfusion on glutathione levels and lipid peroxidation in the liver. Liver reduced glutathione (GSH) (a), oxidized glutathione (GSSG) (b), a ratio of reduced glutathione (GSH) to oxidized glutathione (GSSG) (c), and liver malondialdehyde (MDA) levels (d) were measured in rats subjected to renal IR or sham operation. Results are expressed as mean ± SE (*n* = 4 for each group). ^*∗*^ *p* < 0.05 when compared with the value obtained from the sham-operated group.

**Figure 5:**
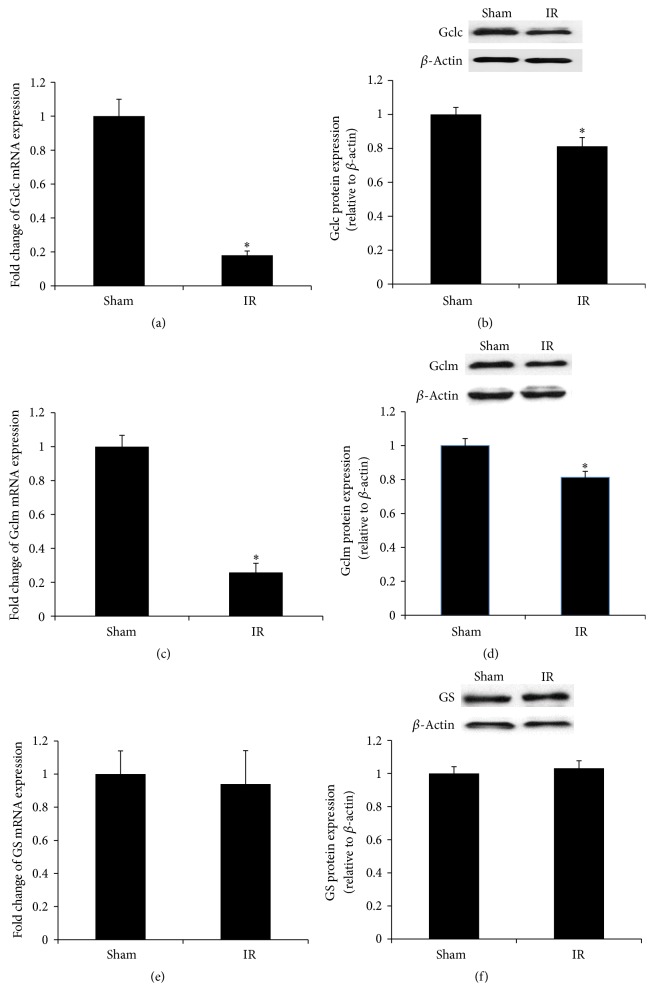
Expression of glutathione synthetic enzymes in the liver. Liver glutamate-cysteine ligase catalytic subunit (*Gclc*) mRNA (a) and protein (b), glutamate-cysteine ligase modifier subunit (*Gclm*) mRNA (c) and protein (d), and glutathione synthase (*GS*) mRNA (e) and protein (f) were measured in rats subjected to renal IR or sham operation. Results are expressed as mean ± SE (*n* = 4 for each group). ^*∗*^ *p* < 0.05 when compared with the value obtained from the sham-operated group.

**Figure 6:**
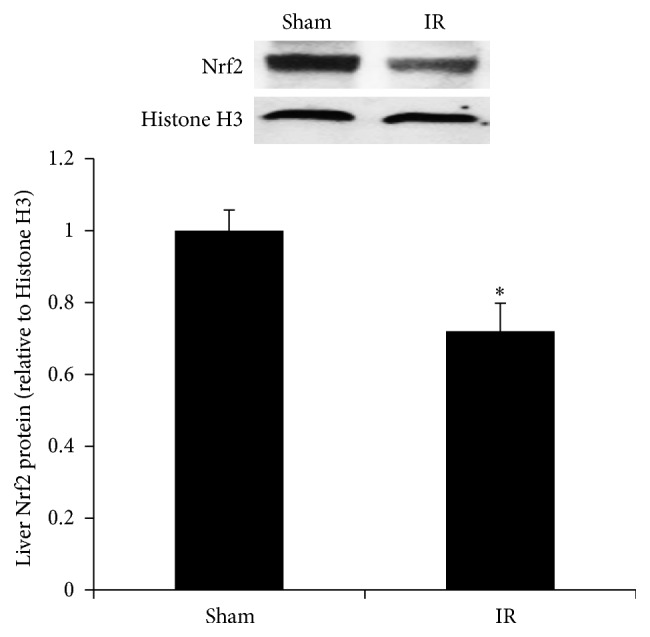
Expression of Nrf2 protein in the liver. The Nrf2 protein was determined by Western immunoblotting analysis of the liver nuclear fraction of rats subjected to renal IR or sham operation. Results are expressed as mean ± SE (*n* = 4 for each group). ^*∗*^ *p* < 0.05 when compared with the value obtained from the sham-operated group.

Renal IR resulted in a significant decrease in GSH levels and a low ratio of GSH to GSSG in the liver (Figures 4(a), 4(b), and 4(c)). The level of MDA, a biomarker for lipid peroxidation, was significantly increased in the liver of rats subjected to renal IR, indicating oxidative stress (Figure 4(d)). To investigate whether low level of glutathione in the liver upon renal IR was due to a decrease in glutathione biosynthesis, we examined the expression of the enzymes that were responsible for its synthesis. Renal IR resulted in a significant decrease in the expression of glutamate-cysteine ligase subunits (*Gclc* and* Gclm*) mRNA and protein in the liver (Figures 5(a), 5(b), 5(c), and 5(d)). However, the expression of glutathione synthase in the liver was not significantly altered by renal IR (Figures 5(e) and 5(f)). The protein level of a transcription factor Nrf2 in the nucleus was significantly lower in the liver of rats subjected to renal IR than that in the sham-operated rats (Figure 6).

### 3.3. Renal Ischemia-Reperfusion Inhibited the Transsulfuration Pathway in the Liver

**Figure 7:**
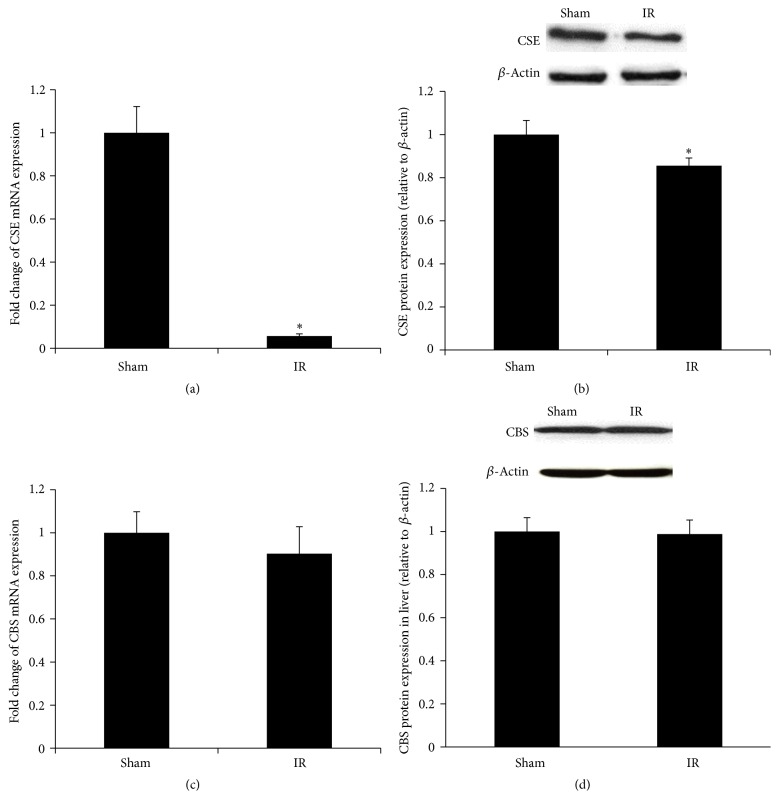
Expression of transsulfuration pathway enzymes in the liver. Cystathionine *γ*-lyase (*CSE*) mRNA (a) and protein (b) and cystathionine *β*-synthase (*CBS*) mRNA (c) and protein (d) were determined in the liver samples of rats subjected to renal IR or sham operation. Results are expressed as mean ± SE (*n* = 4 for each group). ^*∗*^ *p* < 0.05 when compared with the value obtained from the sham-operated group.

**Figure 8:**
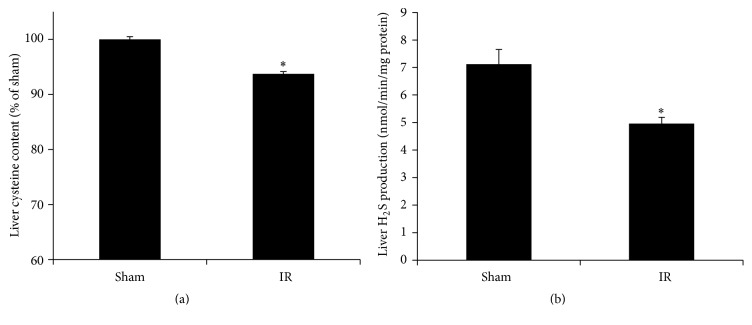
Hepatic cysteine content and hydrogen sulfide production. The cysteine content (a) and hydrogen sulfide (H_2_S) production (b) were measured in the liver of rats subjected to renal IR or sham operation. Results are expressed as mean ± SE (*n* = 4 for each group). ^*∗*^ *p* < 0.05 when compared with the value obtained from the sham-operated group.

The transsulfuration pathway provides cysteine as an essential precursor for glutathione synthesis. To investigate whether renal IR affected this pathway in the liver, the expression of two enzymes (*CBS* and* CSE*) was examined. The mRNA and protein levels of* CSE* were significantly decreased in the liver of rats subjected to renal IR (Figures 7(a) and 7(b)). However, the mRNA and protein levels of* CBS* were not significantly changed (Figures 7(c) and 7(d)). In accordance with a decreased expression of* CSE* in the transsulfuration pathway, the level of hepatic cysteine was significantly lower in rats subjected to renal IR than in the sham-operated group (Figure 8(a)). The production of hydrogen sulfide was significantly reduced in the liver of rats subjected to renal IR (Figure 8(b)).

### 3.4. Regulation of Glutathione Synthesis in HepG2 Cells

**Figure 9:**
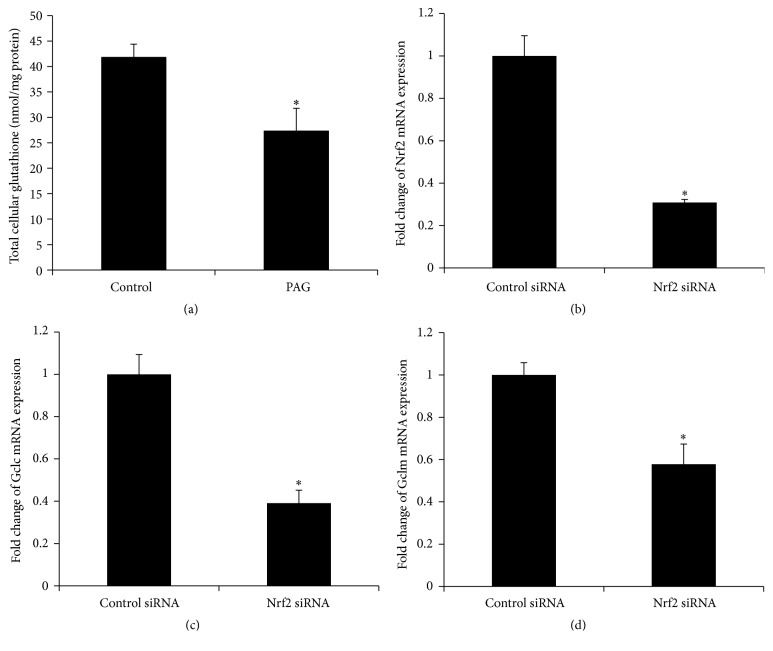
Glutathione measurement and siRNA transfection in HepG2. Cells were incubated in the absence (control) or presence of DL-propargylglycine (PAG) for 16 h and total glutathione was measured (a). Cells were transfected with Nrf2 siRNA or scrambled siRNA (control). The mRNA of Nrf2 (b) and glutamate-cysteine ligase subunits* Gclc* (c) and* Gclm* (d) were determined. Results are expressed as mean ± SE (*n* = 4 for each group). ^*∗*^ *p* < 0.05 when compared with the value obtained from control cells.

To further investigate whether downregulation of hepatic* CSE* expression contributed to decreased glutathione synthesis, experiments were conducted in HepG2 cells. Treatment of cells with a* CSE* inhibitor (DL-propargylglycine, PAG) [39, 47] significantly reduced intracellular glutathione level (Figure 9(a)). Furthermore, transfection of HepG2 cells with Nrf2 siRNA not only inhibited Nrf2 expression (Figure 9(b)) but also significantly reduced mRNA expression of glutamate-cysteine ligase subunits (*Gclc* and* Gclm*) (Figures 9(c) and 9(d)). These results suggested that the transsulfuration pathway and Nrf2 played an important role in regulating glutathione synthesis in hepatocytes.

## 4. Discussion

In the present study, renal IR caused local and distant organ injury which was accompanied by a marked decrease in plasma and hepatic glutathione levels. Depletion of glutathione, a major endogenous nonenzymatic antioxidant, might compromise the ability of the body to cope with oxidative stress locally and systemically. Our study, for the first time, has identified that decreased expression of glutamate-cysteine ligase, a key enzyme for glutathione biosynthesis, and reduced* CSE*-mediated cysteine production through the transsulfuration pathway may be responsible for hepatic glutathione depletion upon renal IR. This, in turn, dampens the antioxidant defense mechanism and contributes to renal IR-induced oxidative stress.

Glutathione serves as a major endogenous antioxidant against oxidative stress. Under physiological conditions, more than 90% of the cellular glutathione pool is in the reduced form (GSH) and less than 10% is in the oxidized (disulfide) form (GSSG). In the present study, the ratio of reduced (GSH) to oxidized (GSSG) glutathione in the liver was markedly decreased in rats subjected to renal IR. The equilibrium between reduced (GSH) and oxidized (GSSG) glutathione reflects the redox potential of a given tissue. Lower GSH to GSSG ratio observed in the liver and plasma indicated that oxidative stress occurred in the distant organs and systemically upon renal IR. This was accompanied by increased hepatic lipid peroxidation. The liver plays an important role in regulating glutathione homeostasis due to its high capacity for glutathione synthesis. However, renal IR caused a significant reduction in hepatic glutathione levels and impaired liver function, which was in line with findings by other investigators [19, 22]. Further investigation revealed that renal IR caused a significant decrease in the expression of hepatic glutamate-cysteine ligase, a key enzyme responsible for glutathione synthesis. Nrf2 is a major transcription factor that induces the expression of antioxidant enzymes including those responsible for glutathione synthesis. Activation of Nrf2 has been implicated as a potential therapeutic target in kidney disease [48–50]. In the present study, renal IR resulted in a significant decrease in Nrf2 protein level in the nucleus. Transfection of hepatocytes with Nrf2 siRNA led to a marked reduction of glutamate-cysteine ligase (*Gclc* and* Gclm*) expression. It is plausible that renal IR might impair Nrf2-dependent glutamate-cysteine ligase expression, which, in turn, led to a decrease in hepatic glutathione synthesis upon renal IR. However, the mechanism by which renal IR leads to a decrease in hepatic nuclear Nrf2 protein remains to be investigated.

The availability of cysteine is another important determinant in modulating glutathione homeostasis as this amino acid is a precursor for glutathione biosynthesis [14, 17, 18]. The transsulfuration pathway catalyzed by two enzymes,* CBS* and* CSE*, is the major source of* de novo* synthesized cysteine in mammalian cells. Relative to other organs,* CBS* and* CSE* are highly expressed in the liver. It has been estimated in murine liver that* CSE* protein levels may be 60 times higher than* CBS* protein [51]. The liver's high rate of transsulfuration activity may contribute to its high capacity for glutathione biosynthesis, compared to other organs [14]. Our results suggested that lower expression of hepatic* CSE* upon renal IR could lead to a decrease in* de novo* cysteine production. This could limit the availability of cysteine and hence decrease glutathione biosynthesis in the liver. Downregulation of* CSE* expression impaired homocysteine metabolism through the transsulfuration pathway, which, in turn, led to a significant elevation of homocysteine levels in the plasma, a condition known as hyperhomocysteinemia. Hyperhomocysteinemia is regarded as a risk factor for cardiovascular disease due to its association with vascular endothelial dysfunction and atherosclerosis [40, 52–54]. We previously reported that elevation of homocysteine levels in the kidney due to downregulation of the transsulfuration pathway directly linked to IR-induced kidney injury [40, 55]. The transsulfuration pathway serves as the source of* de novo* synthesized cysteine in mammalian cells, while cysteine is a precursor for glutathione synthesis. Inhibition of* CBS* and/or* CSE* in the transsulfuration pathway by renal IR can affect cysteine biosynthesis and subsequently glutathione generation. It is plausible that elevated homocysteine levels together with reduced* de novo* cysteine synthesis and glutathione generation may act synergistically in the development of AKI and multiple organ dysfunction. It has been reported that administration of N-acetylcysteine is effective in improving renal function in rats with acute kidney failure [56–58]. Future studies are warranted to examine the effect of N-acetylcysteine/cysteine administration on homocysteine metabolism and distant organ function in AKI. Aside from metabolizing homocysteine to cysteine through the transsulfuration pathway,* CBS *and* CSE* are also responsible for hydrogen sulfide production through desulfuration reactions. In accordance with the low level of* CSE* expression, hydrogen sulfide production was significantly reduced in the liver isolated from rats subjected to renal IR. Hydrogen sulfide is a potent gasotransmitter that has multifaceted effects under both physiological and pathophysiological processes including antioxidant and anti-inflammatory effect and protection of myocardial IR injury [59–62]. Renal IR-induced oxidative stress, hyperhomocysteinemia, and low hydrogen sulfide generation may pose adverse effect to the cardiovascular system.

**Figure 10:**
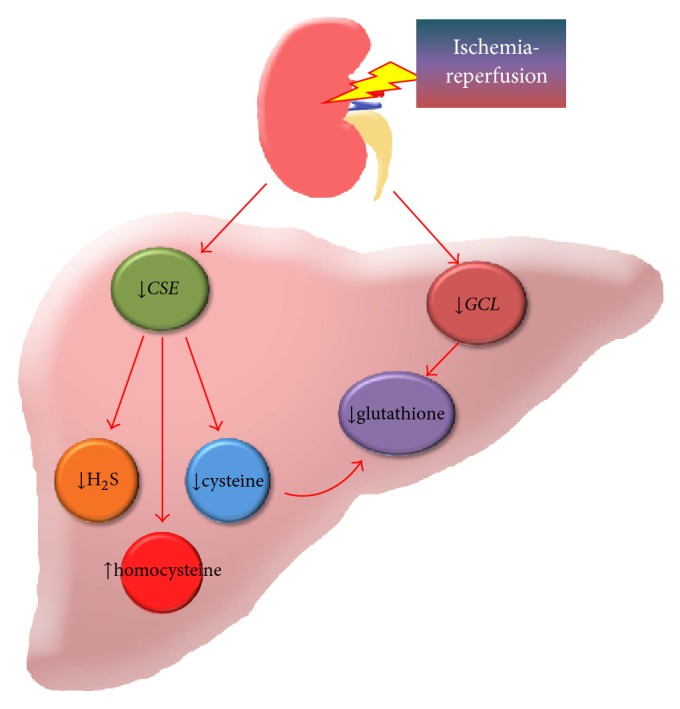
Proposed mechanism of kidney ischemia-reperfusion induced reduction of hepatic glutathione biosynthesis. Renal ischemia-reperfusion (IR) inhibits glutathione biosynthesis in the liver through (1) a decrease in the expression of cystathionine *γ*-lyase (*CSE*) in the transsulfuration pathway which elevates homocysteine and reduces* de novo* cysteine production and hydrogen sulfide (H_2_S) generation and (2) a decrease in the expression of glutamate-cysteine ligase (*GCL*) which is a key enzyme responsible for glutathione biosynthesis.

In conclusion, renal IR elicits liver injury which is accompanied by reduced hepatic production of glutathione, an important endogenous antioxidant. Several lines of evidence obtained from the present study suggest that the remote effect of renal IR on liver glutathione depletion is caused by (1) inhibition of Nrf2-mediated expression of glutamate-cysteine ligase, a key enzyme that regulates glutathione biosynthesis, and (2) downregulation of* CSE* expression in the transsulfuration pathway which limits the availability of cysteine, an essential precursor for glutathione biosynthesis, and elevates homocysteine levels (Figure 10). Such an aberrant response may play a critical role in distant organ injury which, in turn, exacerbates kidney injury. Because the liver has such a high rate of the transsulfuration activity and is the major organ for glutathione biosynthesis, future studies are warranted to investigate whether restoration of enzymes that are responsible for glutathione, homocysteine, and cysteine homeostasis could alleviate AKI-induced oxidative stress and distant organ injury.
